# Epidemiology of Hematologic Malignancies in Real-World Settings: Findings From the Hemato-Oncology Latin America Observational Registry Study

**DOI:** 10.1200/JGO.19.00025

**Published:** 2019-11-27

**Authors:** Vania Tietsche de Moraes Hungria, Carlos Chiattone, Miguel Pavlovsky, Lina M. Abenoza, Gladys P. Agreda, Jorge Armenta, Celso Arrais, Oscar Avendaño Flores, Fernando Barroso, Ana L. Basquiera, Carmen Cao, Maria S. Cugliari, Alicia Enrico, Laura M. Foggliatto, Kenny M. Galvez, David Gomez, Alvaro Gomez, Daniel de Iracema, Danielle Farias, Lineth Lopez, William Armando Mantilla, Deborah Martínez, Maria Jose Mela, Carlos E. Miguel, Roberto Ovilla, Luis Palmer, Carolina Pavlovsky, Christian Ramos, Guillermina Remaggi, Rodrigo Santucci, Sergio Schusterschitz, Claudia Lucia Sossa, Elena Tuna-Aguilar, Jorge Vela, Telma Santos, Odin de la Mora, Gerardo Machnicki, Mariana Fernandez, Paula Barreyro

**Affiliations:** ^1^ Irmandade da Santa Casa de Misericórdia de São Paulo, São Paulo, Brazil; ^2^ Faculdade de Ciencias Médicas–Santa Casa de São Paulo, São Paulo, Brazil; ^3^ FUNDALEU, Buenos Aires, Argentina; ^4^ Fundacion Santa Fe de Bogotá, Bogotá, Colombia; ^5^ Instituto Nacional de Ciencias Médicas y Nutrición Salvador Zubirán, Mexico City, Mexico; ^6^ ISSEMYM, Mexico City, Mexico; ^7^ Hospital Sìrio-Libanês, São Paulo, Brazil; ^8^ Medical Solution SA, Guatemala City, Guatemala; ^9^ Hospital Universitario Walter Cantidio, Fortaleza, Brazil; ^10^ Hospital Privado Centro Médico de Córdoba, Córdoba, Argentina; ^11^ Instituto Nacional del Cancer, Santiago, Chile; ^12^ Instituto de Oncología Ángel H. Roffo, Buenos Aires, Argentina; ^13^ Hospital Italiano La Plata, Buenos Aires, Argentina; ^14^ Hospital das Clinicas de Porto Alegre, Porto Alegre, Brazil; ^15^ Hospital Pablo Tobón Uribe, Medellín, Colombia; ^16^ Hospital Universitario “Dr José E. González,” Mexico City, Mexico; ^17^ Hemato Oncólogos SA, Valle, Colombia; ^18^ CEPHO, Santo André, Brazil; ^19^ Hospital das Clinicas da Universidade Federal de Goiás, Goiânia, Brazil; ^20^ Complejo Hospitalario Metropolitano Dr Annulfo Arias Madrid, Panama City, Panama; ^21^ Fundacion Cardioinfantil, Bogotá, Colombia; ^22^ Fundacao Faculdade Regional de Medicina São José do Rio Preto, São José do Rio Preto, Brazil; ^23^ Hospital Angeles Lomas, Huixquilucan, Mexico; ^24^ Complejo Médico de la PFA Churruca-Visca, Buenos Aires, Argentina; ^25^ Hospital General de México, Mexico City, Mexico; ^26^ Instituto de Ensino e Pesquisas São Lucas, São Paulo, Brazil; ^27^ Hospital das Clinicas–Universidade Federal de Minas Gerais, Belo Horizonte, Brazil; ^28^ Fundacion Oftalmológica de Santander, Santander, Colombia; ^29^ Centro Médico Nacional La Raza, Instituto Mexicano del Seguro Social, Mexico City, Mexico; ^30^ Janssen-Cilag Farmacêutica, São Paulo, Brazil; ^31^ Janssen-Cilag Farmacêutica, Buenos Aires, Argentina

## Abstract

**PURPOSE:**

Limited information is available on multiple myeloma (MM), chronic lymphocytic leukemia (CLL), and non-Hodgkin lymphoma (NHL) management in Latin America. The primary objective of the Hemato-Oncology Latin America (HOLA) study was to describe patient characteristics and treatment patterns of Latin American patients with MM, CLL, and NHL.

**METHODS:**

This study was a multicenter, retrospective, medical chart review of patients with MM, CLL, and NHL in Latin America identified between January 1, 2006, and December 31, 2015. Included were adults with at least 1 year of follow-up (except in cases of death within 1 year of diagnosis) treated at 30 oncology hospitals (Argentina, 5; Brazil, 9; Chile, 1; Colombia, 5; Mexico, 6; Panama/Guatemala, 4).

**RESULTS:**

Of 5,140 patients, 2,967 (57.7%) had NHL, 1,518 (29.5%) MM, and 655 (12.7%) CLL. Median follow-up was 2.2 years for MM, 3.0 years for CLL, and 2.2 years for NHL, and approximately 26% died during the study observation period. Most patients had at least one comorbidity at diagnosis. The most frequent induction regimen was thalidomide-based chemotherapy for MM and chlorambucil with or without prednisone for CLL. Most patients with NHL had diffuse large B-cell lymphoma (DLBCL; 49.1%) or follicular lymphoma (FL; 19.5%). The majority of patients with DLBCL or FL received rituximab plus cyclophosphamide, doxorubicin, vincristine, and prednisone.

**CONCLUSION:**

The HOLA study generated an unprecedented level of high-quality, real-world evidence on characteristics and treatment patterns of patients with hematologic malignancies. Regional disparities in patient characteristics may reflect differences in ethnoracial identity and level of access to care. These data provide needed real-world evidence to understand the disease landscape in Latin America and may be used to inform clinical and health policy decision making.

## INTRODUCTION

Hematologic malignancies (HMs) originate from uncontrolled growth of hematopoietic and lymphoid tissues. These biologically and clinically heterogeneous disorders account for 6.5% of all cancers around the world, including approximately 9.0% in the United States and Europe.^1,2^ Less is known about HM epidemiology in Latin America (Central and South America). The region has witnessed a considerable increase in the amount of hematology and oncology research; however, most of the contributions were observed in just a few countries (Brazil, Argentina, Mexico, Peru, Chile, and Uruguay).^3^

In the multinational CONCORD program, which estimates survival from cancer in 1.9 million adults from 101 population-based cancer registries in 31 countries on 5 continents, only 2 of the participating countries were located in Latin America.^4^ Moreover, according to the WHO, only 8% of Latin American populations are covered by cancer registries.^5^

Real-world data on HMs in Latin America are needed to inform decisions about patient care and health policy. The past 15 years have brought paradigm shifts in diagnosis, staging, and treatment of HMs around the world, but progress in Latin America and the suitability (and potential effects) of new therapies for its residents are largely unknown.^6-13^ The primary aim of the Hemato-Oncology Latin America (HOLA) study was to describe patient characteristics and treatment patterns in individuals with diagnoses of multiple myeloma (MM), chronic lymphocytic leukemia (CLL), or non-Hodgkin lymphoma (NHL) managed at oncology (tertiary care) hospitals in Latin American countries.

**CONTEXT**

**Key Objective**
Despite improved assessment and management of multiple myeloma, chronic lymphocytic leukemia (CLL), and non-Hodgkin lymphoma (NHL) around the world, there is a paucity of high-quality, real-world data on these diseases in Latin America. The aim of the HOLA study is to investigate patient and treatment characteristics to understand the unmet needs in cancer care in Latin America.
**Knowledge Generated**
More than 5,000 patients were included from 7 Latin American countries, with the median age at diagnosis being slightly different from other regions’ reports (range, 57 years in patients with NHL to 67 years in patients with CLL). Most patients had comorbid conditions, and there was considerable regional heterogeneity in characteristics related to both patients and their management.
**Relevance**
Findings from this study can fill an important gap for physicians, patients, and health authorities in Latin America for the management of hematologic malignancies and can be used to understand the disease landscape in this region for further improvement of patient care.

## METHODS

In this multicenter, observational study, the medical records of Latin American patients with CLL, MM, or NHL were retrospectively reviewed (ClinicalTrials.gov identifier: NCT02559583). Adults with HMs were studied at 30 oncology specialty hospitals in 7 countries: Argentina (4 private, 1 public setting), Brazil (1 private, 8 public settings), Chile (1 public setting), Colombia (5 private settings), Mexico (1 private, 5 public settings), Panama (1 private, 2 public settings), and Guatemala (1 public setting). The hospitals were selected based on their experience in providing clinical treatment to patients with CLL, MM, or NHL; geographical representation; the type of practice; interest in participating in the study; and fulfillment of the study requirements. All study sites were eligible to include all 3 types of cancer.

Patient population inclusion criteria were incident or prevalent CLL, MM, or NHL diagnosed between January 1, 2006, and December 31, 2015; age ≥ 18 years at the time of first observed diagnosis of these HMs (whether incident or prevalent); ≥ 1 year of patient data after first observed diagnosis (except in the event of patient death within 1 year of being diagnosed); and ability and willingness to provide informed consent (except if a waiver of informed consent was obtained). There were no exclusion criteria.

The study was conducted under the Guidelines for Good Pharmacoepidemiology Practices issued by the International Society for Pharmacoepidemiology, the Declaration of Helsinki and its amendments, and any applicable national guidelines. Each study was approved by its local ethics committee and followed local country requirements (Brazil, approval by central ethics committee; Colombia, notification to Instituto Nacional de Vigilancia de Medicamentos y Alimentos; Argentina, approval by Direccion Nacional de Protección de Datos Personales; Mexico, approval by Secretaria de Salud/COFEPRIS, Comisión Nacional de Investigación Cientificas; and Guatemala, notification to Ministerio de Salud Publica).

Each study site selected and prepared medical records for chart abstractors to review, and the principal investigator provided clarification in cases of doubt. The study employed chart abstractors with previous experience in performing medical record reviews, and the chart abstractors were centrally trained by Janssen Cilag personnel on the electronic data capture system, study protocol, and how to complete the electronic case report forms (CRFs). The data fields, such as laboratory results, diagnosis specification, and other clinical criteria in the CRFs, had predefined ranges in clickable fields as opposed to free-text fields. This was designed to ensure that the abstractor looked only for the required information in the medical chart and selected the field that contained the specific range in the CRF where the data fit. Each trained chart abstractor signed a training form to document training module completed and date of training. All training materials and records were available for review, and future chart abstractors were required to undergo the same training.under Source document verification visits were performed at selected sites, which represented 60% of all study sites.

Demographic data, including sex, country of residence, age at diagnosis, and age category (< 30, 30 to < 40, 40 to < 50, 50 to < 60, 60 to < 70, 70 to < 80, ≥ 80 years) were collected. Clinical data included comorbidity status, disease stage, Eastern Cooperative Oncology Group (ECOG) performance status (PS), and first-contact physician type at diagnosis. Disease stage at diagnosis was based on International Staging System (ISS) for MM, Ann Arbor staging for NHL, and Binet/Rai stage for CLL. The subtype of NHL was also recorded. Treatment data included the time of treatment initiation and the treatment regimen of the induction therapy. Information about a patient’s transplantation history was also collected from patients diagnosed with MM.

Descriptive statistical analyses were stratified by cancer type and country. Patient characteristics and treatment patterns were summarized using descriptive statistics, including measures of central tendency (mean, median) and spread (variance, range, minimum, maximum) for continuous variables (eg, age at diagnosis) and frequency distributions (number, %) for categorical variables (eg, sex). Because of low overall enrollment, data from Panama and Guatemala were pooled for analysis. Treatment characteristics of patients with MM were further stratified by their history of transplantation (yes/no). Statistical analyses were conducted using SAS 9.2 software (SAS Institute, Cary, NC).

## RESULTS

**FIG 1:**
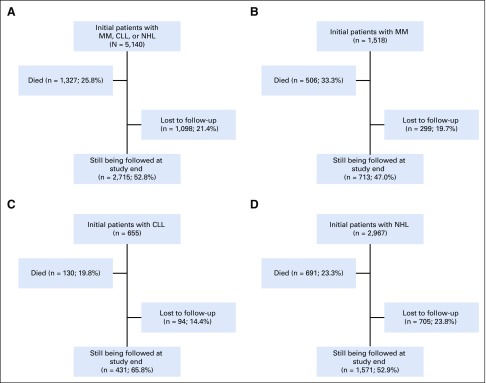
Disposition of (A) all patients and those with (B) multiple myeloma (MM), (C) chronic lymphocytic leukemia (CLL), and (D) non-Hodgkin lymphoma (NHL).

Of a total of 5,140 patients, 2,967 (57.7%) had NHL, 1,518 (29.5%) had MM, and 655 (12.7%) had CLL. Among the original 5,140 patients, 2,715 (52.8%) were still being followed at the study end date (Fig 1).

### MM

Among 1,518 initially eligible individuals with MM, 713 (47.0%) were still being followed at the time of study end, with a median follow-up of 2.2 years (range, < 0.1-12.1 years). One third of the patients with MM (506; 33.3%) died during the observation period (range, 18.4% in Panama/Guatemala to 54.4% in Brazil; Table 1). The median age at diagnosis was 61 years (range, 23-91 years). The most frequent age categories were 60 to < 70 years (31.1%) and 50 to < 60 years (28.3%). There was a slight predominance of males (50.7%) over females (49.3%), and the sex balance varied across regions (Table 2).

**TABLE 1:**
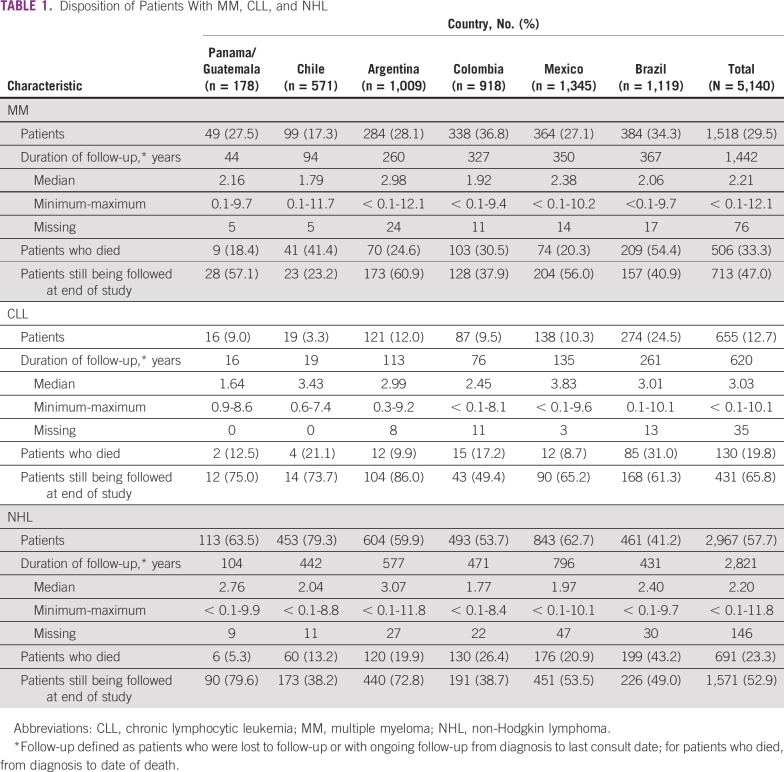
Disposition of Patients With MM, CLL, and NHL

**TABLE 2:**
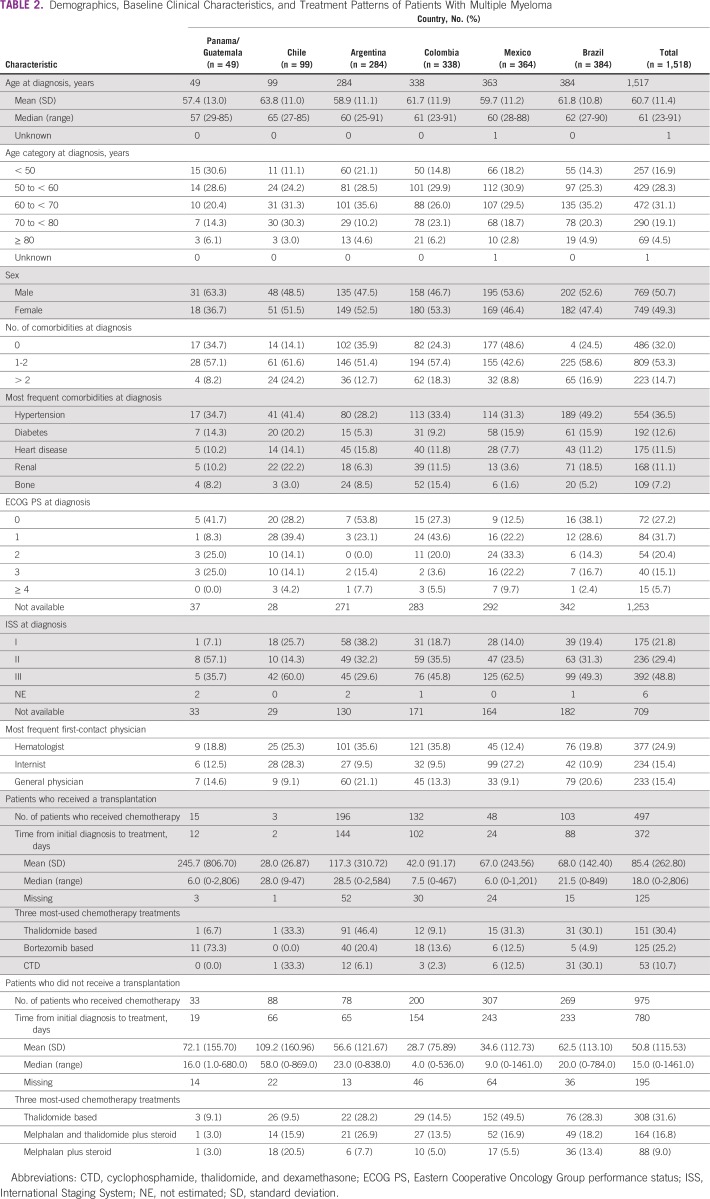
Demographics, Baseline Clinical Characteristics, and Treatment Patterns of Patients With Multiple Myeloma

MM was highly comorbid with a range of chronic and other conditions, with approximately 53% of patients with one or two comorbidities and approximately 15% with more than two comorbidities. Hypertension was reported most frequently in 36.5% of patients (range, 28.2% in Argentina to 49.2% in Brazil). Other frequent comorbidities were diabetes and heart disease in 12.6% and 11.5% of patients, respectively. Potential manifestations of target organ damage included a history of bone disorders in 7.2% of patients and renal disease in 11.1% (Table 2).

At diagnosis, patients had an ECOG PS of 0 (27.2%), 1 (31.7%), or 2 (20.4%). Most patients were ISS stage II (29.4%) or III (48.8%). Stage III MM was the most frequently observed disease stage in Mexico (62.5%), Chile (60.0%), Brazil (49.3%), and Colombia (45.8%). Most Panamanian/Guatemalan patients (57.1%) had stage II, and most Argentinean patients had stage I (38.2%). Of patients with MM, 35% had renal impairment, which was fairly constant across countries. Of all patients with MM, first physician contact was with hematologists in 24.9% followed by 15.4% each with internists and general physicians.

In the entire cohort, 497 patients with MM (32.7%) underwent autologous hematopoietic stem-cell transplantation (ASCT); however, the proportion of patients who underwent transplantation varied among countries. The highest rate was 69.0% in Argentina, and the lowest was 3.0% in Chile. Median time from diagnosis to initial treatment was 18.0 days (range, 6.0 days in Panama/Guatemala and Mexico to 28.5 days in Argentina). The 497 transplantation recipients who received induction chemotherapy were treated predominantly with thalidomide-based (151; 30.4%) and bortezomib-based (125; 25.2%) regimens. In these patients, median time from diagnosis to initial treatment was 15.0 days (range, 4.0 days in Colombia to 58.0 days in Chile). Most patients with MM who did not undergo transplantation received thalidomide-based chemotherapy (308 of 975; 31.6%) or melphalan, thalidomide, and steroid treatment (164 of 975; 16.8%) at induction. Thalidomide-based chemotherapy was the preferred treatment in Argentina (28.2%), Mexico (49.5%), and Brazil (28.3%).

### CLL

Among 655 initially eligible individuals with CLL, 431 (65.8%) were still being followed at the time of study end, with a median follow-up of 3 years (< 0.1-10.1 years; Table 1). The proportion of patients with CLL who died during the observation period was 19.8% (range, 8.7% in Mexico to 31.0% in Brazil). Median age at diagnosis was 67 years (range, 23-96 years), which was relatively consistent across countries (Table 2). The most frequent age categories were 60 to < 70 years (34.6%) and 70 to < 80 years (29.1%). There was a slight predominance of male (54.2%) over female (45.8%) patients with CLL.

A total of 468 (71.5%) of the 655 patients with CLL had comorbidities at diagnosis (Table 3). The most frequent comorbidity was hypertension observed in 46.1% (range, 37.5% in Panama/Guatemala to 73.7% in Chile); other common comorbidities were heart disease (15.7%) and diabetes (15.1%).

**TABLE 3:**
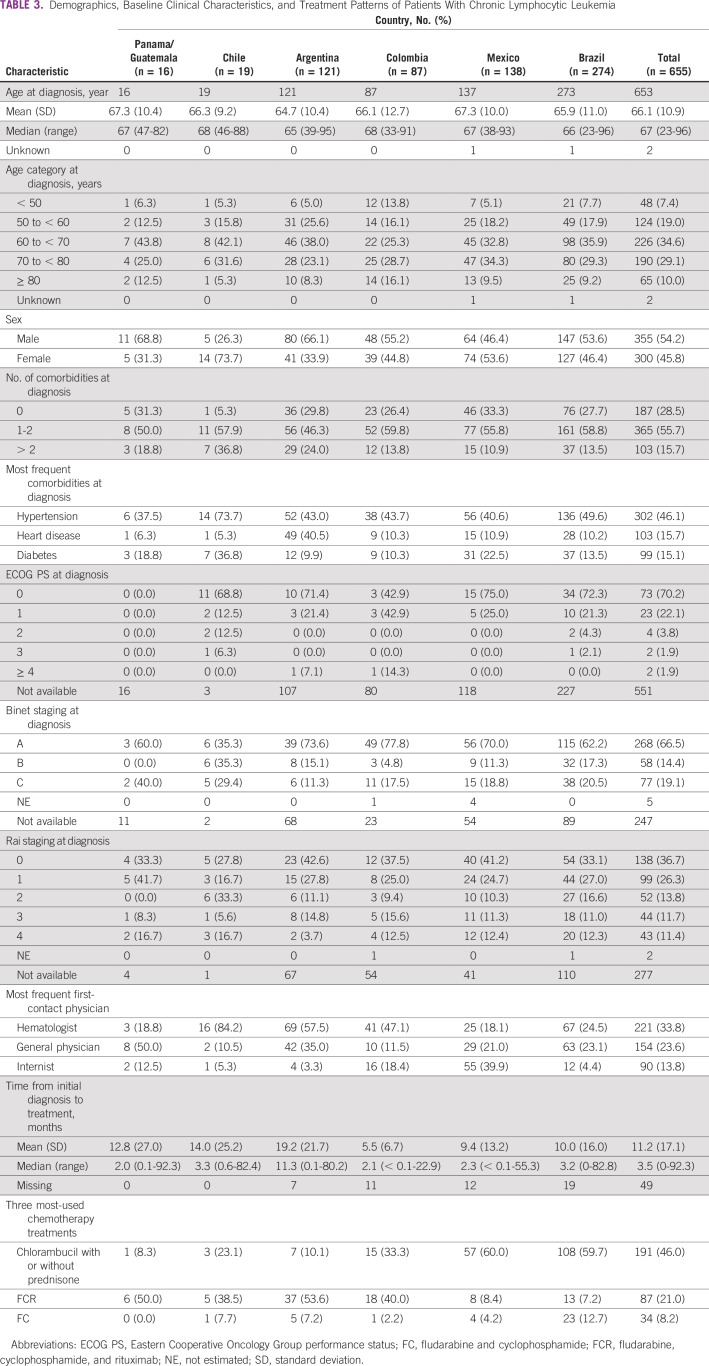
Demographics, Baseline Clinical Characteristics, and Treatment Patterns of Patients With Chronic Lymphocytic Leukemia

Approximately two thirds of patients with CLL presented with Binet stage A (66.5%) and/or Rai stage 0 or 1 (63.0%). Most patients (92.3%) had an ECOG PS of 0 or 1 (range, 81.3% in Chile to 93.6% in Brazil) at diagnosis. Compared with other specialties, the first physician contacted most frequently was with a hematologist (33.8%; range, 18.1% in Mexico to 84.2% in Chile). Other physicians were general physicians (23.6%) and internists (13.8%).

Of the 655 patients with CLL, 415 (63.4%) received chemotherapy. The most common initial treatment of CLL was chlorambucil with or without prednisone (191; 46.0%) followed by fludarabine, cyclophosphamide, and rituximab (FCR; 87; 21.0%) and fludarabine and cyclophosphamide (34; 8.2%). The majority of patients in Brazil (108 of 181; 59.7%) and Mexico (57 of 95; 60.0%) received chlorambucil with or without prednisone. FCR was the most frequent initial chemotherapy regimen in Argentina (53.6%), Panama/Guatemala (50.0%), Colombia (40%), and Chile (38.5%).

### NHL

Among 2,967 eligible individuals with NHL, 1,571 (52.9%) were still being followed at the time of study end, with a median follow-up of 2.2 years (< 0.1-11.8 years). The proportion of patients with NHL who died during the observation period was 23.3% (range, 5.3% in Panama/Guatemala to 43.2% in Brazil; Table 1). More than two thirds of patients with NHL (68.6%) had either diffuse large B-cell lymphoma (DLBCL; 1,457; 49.1%) or follicular lymphoma (FL; 578; 19.5%). This section focuses on these subpopulations. Additional descriptive data on individuals with mantle cell lymphoma and mucosa-associated lymphoid tissue (MALT) lymphoma are listed in Appendix Tables A1 and A2.

Median age at diagnosis was 58 years (range, 18-95 years) for DLBCL and 57 years (range, 18-92 years) for FL. These values showed considerable variation across countries. Sex was approximately evenly distributed in patients with DLBCL and slightly imbalanced toward females with FL (Tables 4 and 5).

**TABLE 4:**
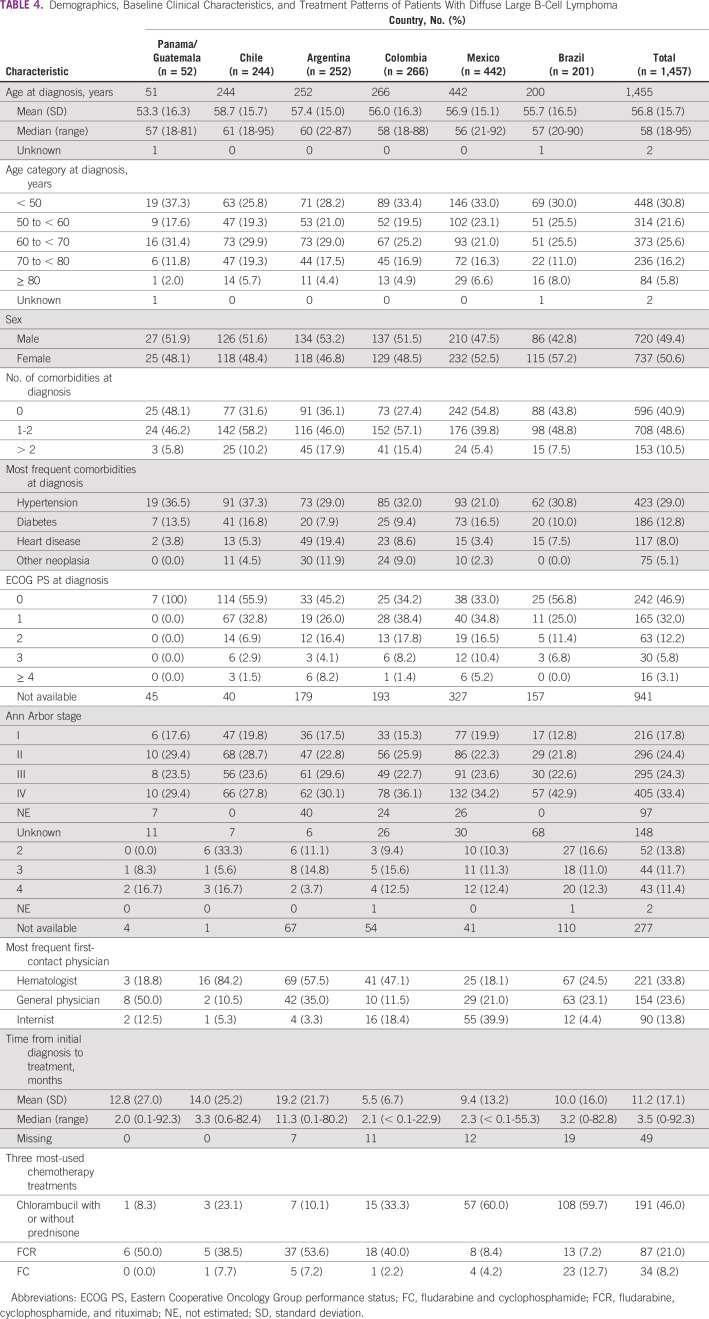
Demographics, Baseline Clinical Characteristics, and Treatment Patterns of Patients With Diffuse Large B-Cell Lymphoma

**TABLE 5:**
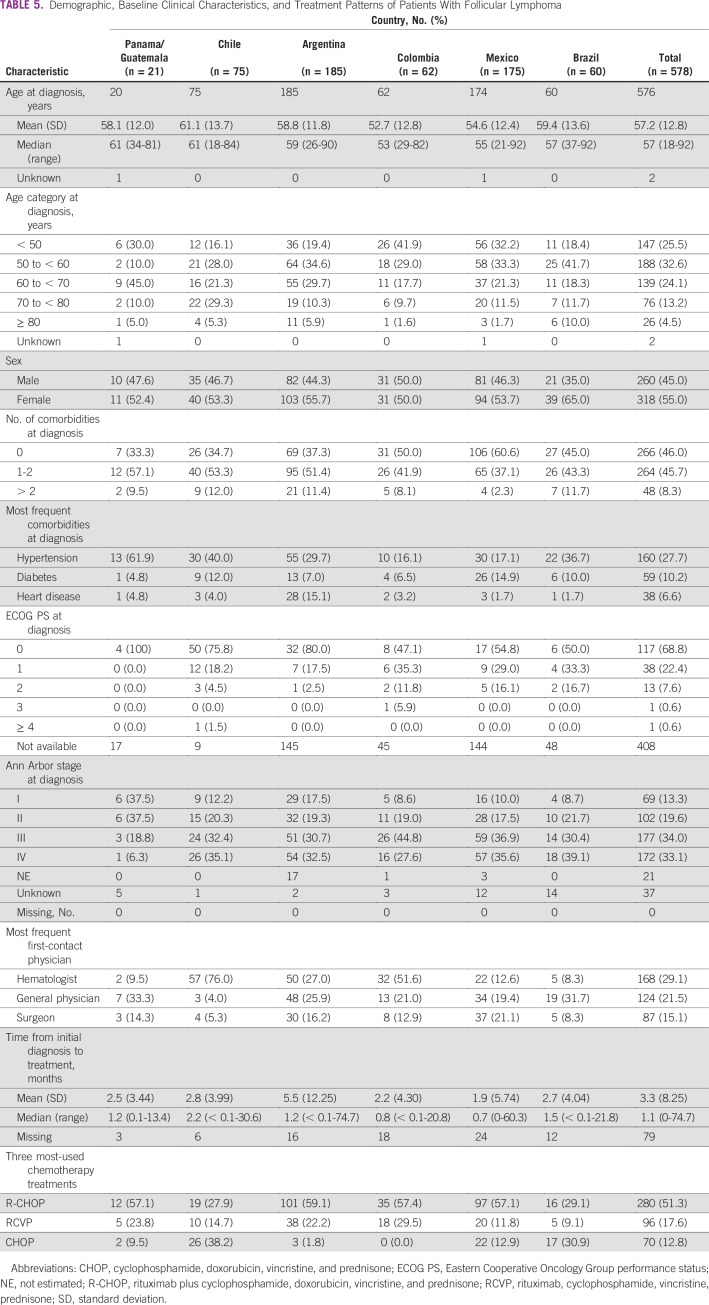
Demographic, Baseline Clinical Characteristics, and Treatment Patterns of Patients With Follicular Lymphoma

Comorbidities were present in 59.1% of patients with DLBCL and 54.0% with FL. The most frequent comorbidity for patients with DLBCL was hypertension (29.0%) followed by diabetes (12.8%) and heart disease (8.0%). For patients with FL, the most frequent comorbidity was hypertension (27.7%) followed by diabetes (10.2%) and heart disease (6.6%).

At diagnosis, patients with DLBCL had an ECOG PS of 0 (46.9%) or 1 (32.0%). Approximately one third of patients with DLBCL (33.4%) who had available Ann Arbor disease staging data were classified as having stage IV; 24.4% had stage II disease, 24.3% had stage III, and 17.8% had stage I. A total of 29.4% of patients with FL had an ECOG PS, of whom 68.8% had a PS of 0 and 22.4% had a PS of 1. A total of 520 (90.0%) of 578 patients with FL had available staging data, with 33.1% having stage IV, 34.0% stage III, 19.6% stage II, and 13.3% stage I disease.

Among patients with DLBCL and FL, hematologists were most frequently the first physician contacted (28.9%) followed by internists/general physicians (21.0%) and surgeons (13.7%). Only 8%-12% of patients in Brazil and Panama/Guatemala first consulted with a hematologist.

For patients with DLBCL, the 2 most frequent regimens were rituximab plus cyclophosphamide, doxorubicin, vincristine, and prednisone (R-CHOP; 70.3%) and cyclophosphamide, doxorubicin, vincristine, and prednisone (CHOP; 14.6%). All other regimens were used by < 5% of patients. Similar trends were observed across countries. The majority of patients with FL received chemotherapy (546 of 578; 94.5%); the 3 most frequent regimens were R-CHOP (280 of 546; 51.3%), rituximab plus cyclophosphamide, vincristine, and prednisone (96 of 546; 17.6%), and CHOP (70 of 546; 12.8%). CHOP was the most frequently reported therapy in Chile (26 of 68; 38.2%) and Brazil (17 of 55; 30.9%).

## DISCUSSION

To our knowledge, the HOLA registry—unprecedented in size and scope—provides high-quality, real-world data from > 5,000 patients with HMs observed for 8 years in Latin America. Despite considerable heterogeneity across the 7 participating countries, certain common themes are of potential concern to health care providers and policymakers. First, large proportions of patients with HMs had comorbidities, which can limit therapeutic options. Second, considerable numbers of patients also had high-grade and other aggressive forms of disease (eg, DLBCL), and approximately 26% of patients died during the observation period.

Our study identified potential regional access-to-care issues. Between 28% and 50% of patients with MM received thalidomide-based chemotherapy in Argentina, Brazil, Mexico, and Chile, and 36% of those in Colombia received bortezomib. Furthermore, there was wide variation between countries in the proportion of patients with MM who had undergone transplantation. In Argentina, 20-fold more patients underwent ASCT than in Chile. The treatment regimens in Latin American were different compared with those of other countries in the world. For example, the majority of transplantation recipients with MM were administered thalidomide-based (30.4%) or bortezomib-based (25.2%) treatment in the studied regions, while lenalidomide, bortezomib, and dexamethasone chemotherapy is the most commonly used frontline treatment of transplantation-eligible patients with MM in the United States. Differences in treatment regimens were probably due to the restricted access and lack of approved novel agents in these studied regions.

With regard to comorbidities, these included hypertension (range across HMs, 29.0%-46.1%), diabetes (12.6%-15.1%), and heart disease (8.0%-15.7%). In one study, patients with MM who received treatment with thalidomide experienced a 23% increase in grade 3-4 adverse events, including, most frequently, cardiovascular disease followed by other hematologic conditions, thromboembolic events, infection, and neuropathy.^14^

A Latin American observational study found the median age of patients with MM to be 67.4 years, and 70.7% studied were in ISS stage II or III. Approximately one third of patients received thalidomide as frontline therapy, and 26.9% received ASCT.^15^ An analysis of medical records for 9,120 patients at a hematologic clinic in Puebla, Mexico, over 20 years identified 855 individuals with HMs, including 66 (7.7%) with MM; median age was 66 years, and 51% survived through 540 days. The authors contended that a range of immunoproliferative disorders are less frequent in the indigenous Mexican population (mestizos) compared with whites, including CLL (5 times less frequent) and monoclonal gammopathy of undetermined significance (4 times less frequent).^16^

In a series of 1,028 patients with NHL in Central America (Guatemala) and South America (Argentina, Brazil, Chile, and Peru), Laurini et al^17^ had findings consistent with those of the HOLA study. Forty percent of patients with NHL had DLBCL. In the HOLA study, 49.1% of patients with NHL had DLBCL (63.5% in Panama/Guatemala). In all, 20.4% of Central and South American patients with NHL had FL; 6.9% had marginal zone B-cell lymphoma, MALT type; and 6.8% had peripheral T-cell lymphomas. Median age of Latin American patients with NHL was 59 years compared with 58 (in patients with DLBCL) in the HOLA study. Other studies in Guatemala,^17^ Mexico,^18^ and Chile^19^ echo the findings from the HOLA study, showing that DLBCL was the most frequent subtype of NHL and revealing that most patients had a B-cell rather than a T-cell lineage. The Guatemalan study of 226 consecutive biopsy samples showed that 44% of patients with NHL had DLBCL (median age, 58.5 years).^17^

The combination of the HOLA cohort size (N = 5,140) and broad scope of follow-up data (8 years) are study strengths. Given the liberal eligibility criteria and observational nature of the study, the findings should be generalizable to most Latin American practitioners’ treatment populations and settings. Chart abstractors were centrally trained and had considerable expertise in using clearly prespecified disease definitions. On the other hand, the cohort represented a convenience population, which potentially reduces the generalizability of the findings. Because of potential medical surveillance bias, the prevailing tertiary care nature of the treatment milieus may have resulted in overestimations of the percentage of patients with HMs, comorbidities, and treatment patterns compared with less-specialized clinics. Given that Latin American cancer care delivery systems are largely skewed toward urban settings, individuals who reside in rural locales may have been under-represented. Rural workers may experience greater vocational exposure to insecticides and other lymphomagens.^20^ The International Classification of Diseases for Oncology codes used to identify patients with MM, CLL, and NHL were developed for reimbursement, not for case ascertainment purposes. Numbers of centers, and hence overall denominators in calculations, were somewhat small, especially in Chile and Panama/Guatemala.

In conclusion, the HOLA study generated an unprecedented level of high-quality, real-world evidence on the disease and treatment characteristics of patients with HMs. Considerable regional variations in HM management were observed, and the findings can likely be ascribed to heterogeneity in both patient characteristics and treatment patterns. Results from this study can be used to understand the disease landscape in Latin America. Future research is needed to explicitly associate observed trends with treatment outcomes.

## Figures and Tables

**TABLE A1:**
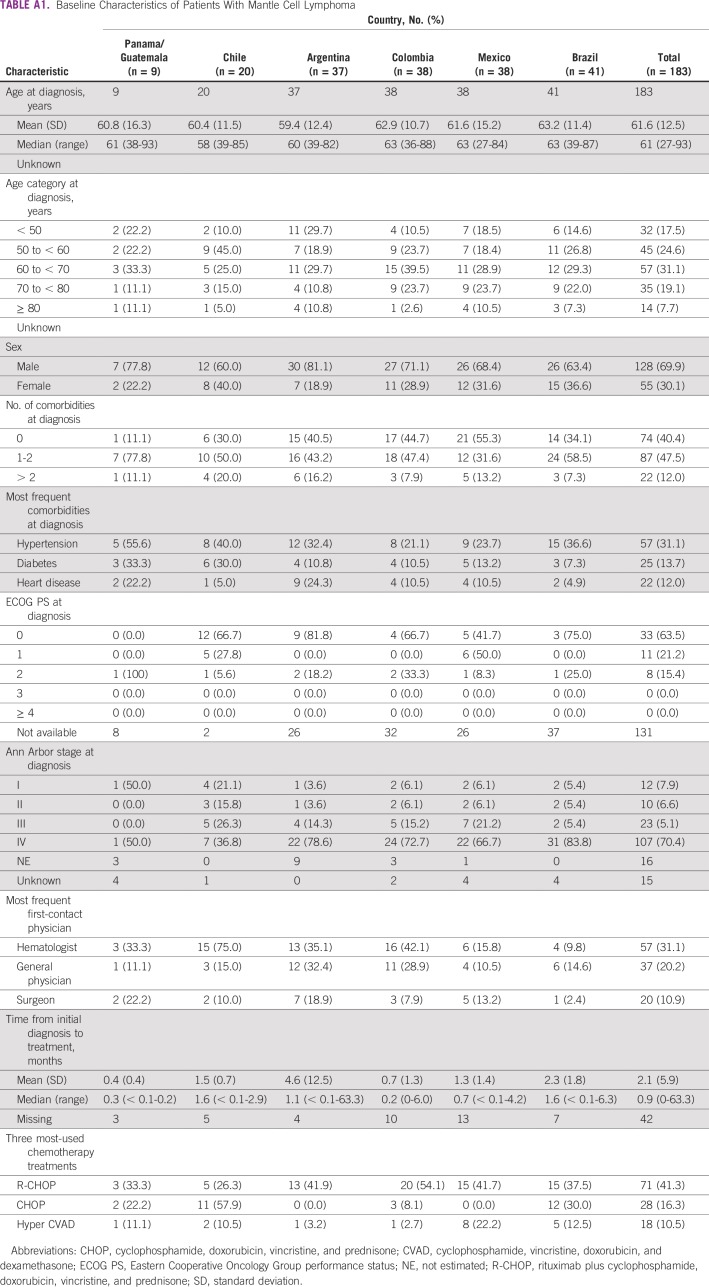
Baseline Characteristics of Patients With Mantle Cell Lymphoma

**TABLE A2:**
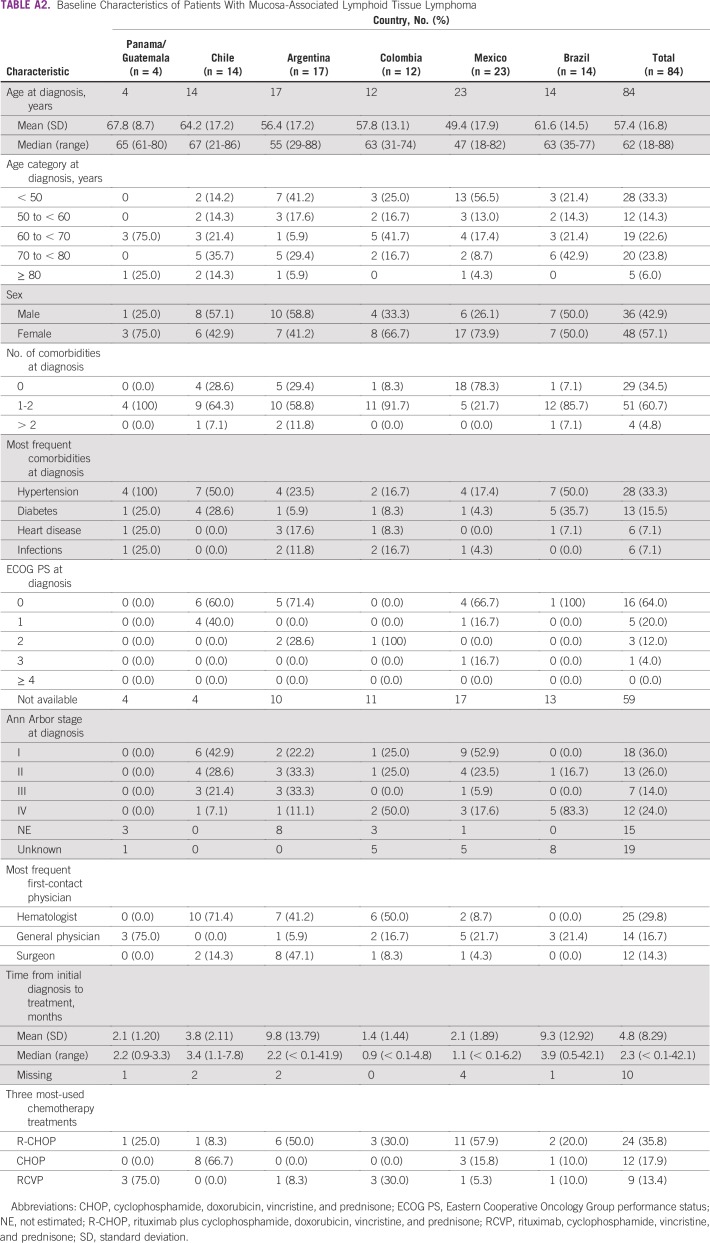
Baseline Characteristics of Patients With Mucosa-Associated Lymphoid Tissue Lymphoma
